# Outcomes and recurrence pattern analysis of intensity modulated chemoradiotherapy in nasopharyngeal cancer: a retrospective study from Heidelberg University Hospital

**DOI:** 10.1186/s13014-025-02769-7

**Published:** 2025-12-08

**Authors:** Lukas Bauer, Sebastian Regnery, Maximilian Y. Deng, Florian Stritzke, Philipp Schröter, Henrik Franke, Nils B. Netzer, Kristin Uzun-Lang, Katharina Weusthof, Rubens Thoelken, Jürgen Debus, Thomas Held

**Affiliations:** 1 Department of Radiation Oncology, Heidelberg University Hospital, Medical Faculty Heidelberg, Heidelberg University, Im Neuenheimer Feld 400, 69120 Heidelberg, Germany; 2 Heidelberg Institute of Radiation Oncology (HIRO), Heidelberg, Germany; 3 National Center for Tumor Diseases (NCT), Heidelberg, Germany; 4 Heidelberg Ion-Beam Therapy Center (HIT), Department of Radiation Oncology, Heidelberg University Hospital, Heidelberg, Germany; 5 Clinical Cooperation Unit Radiation Oncology, German Cancer Research Center (DKFZ), Heidelberg, Germany; 6 Department of Otorhinolaryngology, Heidelberg University Hospital, Heidelberg, Germany

**Keywords:** Nasopharyngeal carcinoma, Intensity-modulated radiotherapy, Treatment failure patterns, Toxicity, Locoregional recurrence

## Abstract

**Background:**

To evaluate treatment outcomes, toxicity, and recurrence patterns by dose level in nasopharyngeal carcinoma (NPC) patients treated with intensity-modulated radiotherapy (IMRT) and weekly cisplatin.

**Methods:**

We retrospectively analyzed 48 NPC patients treated between 2005 and 2019 with IMRT and weekly cisplatin (40 mg/m²). The planning target volume (PTV) received a median total dose of 57.6 Gy (1.8 Gy/fraction) with a simultaneous integrated boost to the primary tumor and nodal metastases up to 70.4 Gy. To assess recurrence patterns, follow-up imaging was deformably co-registered with planning CTs (pCT), and recurrent gross tumor volumes (rGTVs) were delineated and mapped to pCTs. Recurrences were categorized using a centroid-based system into five types: A (central high-dose), B (peripheral high-dose), C (central intermediate-/low-dose), D (peripheral intermediate-/low-dose), and E (extraneous dose).

**Results:**

With a median follow-up of 73 months (range 24–156), 9 patients (19%) had died. The 3-, 5-, and 10-year overall survival rates were 98%, 96%, and 67%, respectively. Local control rates (LCR) at 2, 3, and 5 years were 92%, 89%, and 89%; regional control was 96%, 94%, and 94%; and distant control was 92%, 89%, and 89%. Treatment was well tolerated, with no grade ≥ 4 toxicities. Grade 3 acute toxicities occurred in 23 patients (48%), most commonly dysphagia, with nearly all resolving within 90 days. Among treatment failures, distant metastases (13%) and local relapses (10%) were most frequent. Of 8 local and/or regional recurrences analyzed, 2 were type A (central high-dose), 3 type B (“marginal”), 2 type C (central intermediate-/low-dose), and 1 type E (“out-of-field”).

**Conclusion:**

IMRT with weekly cisplatin yields excellent survival and locoregional control with acceptable toxicity in NPC. Distant metastasis as one of the predominant failure patterns highlights the need for more effective systemic therapies. Most local recurrences arose within high-dose areas, suggesting a potential opportunity for treatment optimization.

## Background

Nasopharyngeal carcinoma (NPC) is a relatively rare malignancy, accounting for less than 1% of all cancers worldwide, with an estimated 133,000 new cases and 80,000 deaths annually [1]. Histologically, NPC is classified into three subtypes by the World Health Organization (WHO): keratinizing squamous cell carcinoma (WHO type I), non-keratinizing squamous cell carcinoma (WHO type II), and basaloid squamous cell carcinoma (WHO type III) [2]. Prognosis is influenced by various factors, including TNM classification, WHO type, Epstein-Barr virus (EBV) association, age, and the expertise of the treatment facility [3].

NPC is a radiosensitive malignancy with an anatomical location that often restricts surgical treatment options. Consequently, radiotherapy serves as the cornerstone of treatment across all stages of the disease. The Union for International Cancer Control (UICC) and the American Joint Committee on Cancer (AJCC) classify NPC into five stages (I–IVB), each requiring distinct therapeutic approaches [4]. According to National Comprehensive Cancer Network (NCCN) and European Society for Medical Oncology (ESMO) guidelines, radiotherapy alone for stage I disease is recommended [5–7]. For stage II disease, concomitant chemotherapy should be considered. For locoregionally advanced disease (UICC III–IVA), recommendations diverge. According to NCCN guidelines, induction chemotherapy followed by concurrent chemoradiotherapy is the preferred strategy, whereas chemoradiotherapy alone carries a category 2B recommendation. Historically, earlier ESMO guidelines did not recommend adjuvant or induction chemotherapy as first choice [8, 9]. In contrast, the more recent ESMO guideline endorses both chemoradiotherapy alone and induction chemotherapy followed by chemoradiotherapy as level IA options for most of stage III–IVA NPC. If induction chemotherapy is not used, both guidelines recommend considering adjuvant chemotherapy.

Regarding radiotherapy technique, IMRT alone has led to significant improvements in outcomes and treatment-related side effects compared to two- or three-dimensional conformal radiotherapy [10–13]. Despite advancements in IMRT, recurrence remains a significant challenge, with both in-field and distant metastases contributing to treatment failure [14, 15]. Large-scale retrospective studies have shown that in-field recurrence is the most common pattern, occurring in 75–93% of relapsed cases, particularly within the nasopharynx and skull base [14, 16]. A study analyzing 645 NPC patients found that most recurrences occur within the first three years after treatment, with in-field failure accounting for 93.3% of local relapses [14]. Another analysis of 869 patients emphasized the importance of individualized clinical target volume (CTV) delineation, as recurrence patterns followed specific anatomical pathways, particularly through neural foramina and ipsilateral cavernous sinuses [17]. Very recent multi-society, evidence-based consensus updates reflect that evolving patterns of failure and stepwise routes of spread necessitate continual refinement and harmonization of target delineation in NPC [18, 19].

The continual refinement of IMRT has not only improved precision in target volume delineation and dose administration but has also facilitated the advancement of more sophisticated methods for recurrence pattern analysis [20–22]. Unlike conventional three-dimensional radiotherapy (3D-RT), where recurrence classification based on anatomical or field-based categorizations - such as in-field, marginal, or out-of-field - was generally adequate, the complexity of IMRT, with its highly conformal and heterogeneous dose distributions, necessitates a more nuanced approach. Traditional classification methods may obscure critical insights into the underlying mechanisms of treatment failure. A recently developed methodology integrating spatial and dosimetric analysis of recurrence sites offers a more comprehensive framework for classifying failure patterns by correlating them with their likely origins [21, 23]. To our knowledge, this methodology has not previously been used to analyze locoregional failure patterns after chemoradiotherapy of patients with NPC. Applying this approach to a cohort of nasopharyngeal carcinoma (NPC) patients treated with chemoradiotherapy may yield valuable insights into dose-specific recurrence trends, thereby enhancing the understanding of treatment failure and informing future therapeutic strategies.

In this study, we conducted a comprehensive analysis of treatment outcomes, toxicity, and recurrence patterns in patients with NPC who underwent chemoradiotherapy with IMRT at the Department of Radiation Oncology, Heidelberg University Hospital, between 2005 and 2019. Utilizing an integrated spatial and dosimetric analysis approach, we aimed to provide a detailed assessment of recurrence patterns and their relationship to treatment parameters.

## Methods

### Screening

A total of 112 patients diagnosed with NPC were identified from the cancer registry of our clinic. This retrospective analysis included all patients who underwent primary radiotherapy using IMRT at our institution. Patients with adenoid cystic carcinoma (ACC), those who received adjuvant or additive radiotherapy, or those treated with protons or carbon ions were excluded. Between 2005 and 2019, a total of 48 patients were treated with IMRT at our clinic.

### Patient characteristics

In total, 48 patients (75% males) with a median age of 55 years receiving IMRT for NPC were included in the retrospective analysis. Before initiation of radiotherapy, staging was conducted using the eighth edition of the Union for International Cancer Control (UICC) TNM system. Most of the patients showed advanced stages of NPC with T4 (18 patients, 38%), N2/3 (31 patients, 64%) and UICC stage III (22 patients, 46%) or IVa (20 patients, 42%). EBV status was unknown in 50% of the patients. Among those with available EBV data, 16 tumors tested positive for EBV. The most frequently observed histological subtype was non-keratinizing squamous cell carcinoma, which was identified in 44 patients (92%). Detailed patient characteristics are described in Table 1.

**Table 1:** Patient characteristics

Parameter	Patients (%)
**Median age**	55 (25–84) years
**Sex**	
Male	36 (75)
Female	12 (25)
**EBV status**	
Positive	16 (33)
Negative	8 (17)
Unknown	24 (50)
**UICC stage**	
I	3 (6)
II	3 (6)
III	22 (46)
IVa	20 (42)
**TNM stage**	
T1	6 (12)
T2	12 (25)
T3	12 (25)
T4	18 (38)
N0	10 (21)
N1	7 (15)
N2	28 (58)
N3	3 (6)
**Histology**	
Keratinizing	3 (6)
Nonkeratinizing	44 (92)
Basaloid	1 (2)

### Treatment features

Immobilization of patients was done using a thermoplastic head-mask system with shoulder fixation. Treatment planning was based on computed tomography (CT) scans with 3.0 mm slice thickness and contrast-enhanced magnetic resonance imaging (MRI). Target volumes were defined according to standard procedures at our institution. The clinical target volume 1 (CTV1) consisted of the visible tumor (gross tumor volume, GTV), involved lymph nodes and a 5 mm safety margin. While CTV2 included CTV1 with a 5 mm safety margin, it also encompassed the typical pathways of tumor spread and elective nodal levels, as defined by the guidelines of Biau et al., with modifications based on the primary tumor site, tumor size, and nodal status [24]. Planning target volumes (PTVs) were generated by applying a 3-mm margin around the CTVs. However, in cases where this expansion would compromise critical structures, such as the optic system, the margin was either reduced or omitted as necessary.

Cumulative median total dose of IMRT of CTV2/PTV2 was 57.6 Gy in a single dose of 1.8 Gy including a simultaneous integrated boost (SIB) of the primary tumor and lymph node metastases (CTV1/PTV1) up to a cumulative median total dose of 70.4 Gy in a single dose of 2.2 Gy in 5 fractions/week.

Concomitant chemotherapy with cisplatin at a dose of 40 mg/m² body surface area was administered weekly in the absence of contraindications. Only one patient received adjuvant chemotherapy following the Al-Sarraf protocol, consisting of cisplatin and 5-fluorouracil.

### Follow-up

Patients’ follow-up examinations were scheduled every three months during the first two years after the end of radiotherapy, every six months during the following two years and then once a year with contrast-enhanced MRI or CT scans of the head and neck. CT scans of the chest and ultrasound of the abdomen were performed annually. A radiation oncologist recorded current symptoms and toxicities related to treatment at each follow-up visit. Clinical examination by an ear, nose and throat specialist was performed regularly. Evaluation of toxicities related to treatment was done from medical records using the Common Terminology Criteria for Adverse Events (CTCAE) version 5. Alongside the standard toxicity assessment conducted according to CTCAE version 5, the average number of toxicity events per patient was analyzed using the TAME methodology [25].

### Analysis of locoregional recurrence patterns

**Fig. 1:**
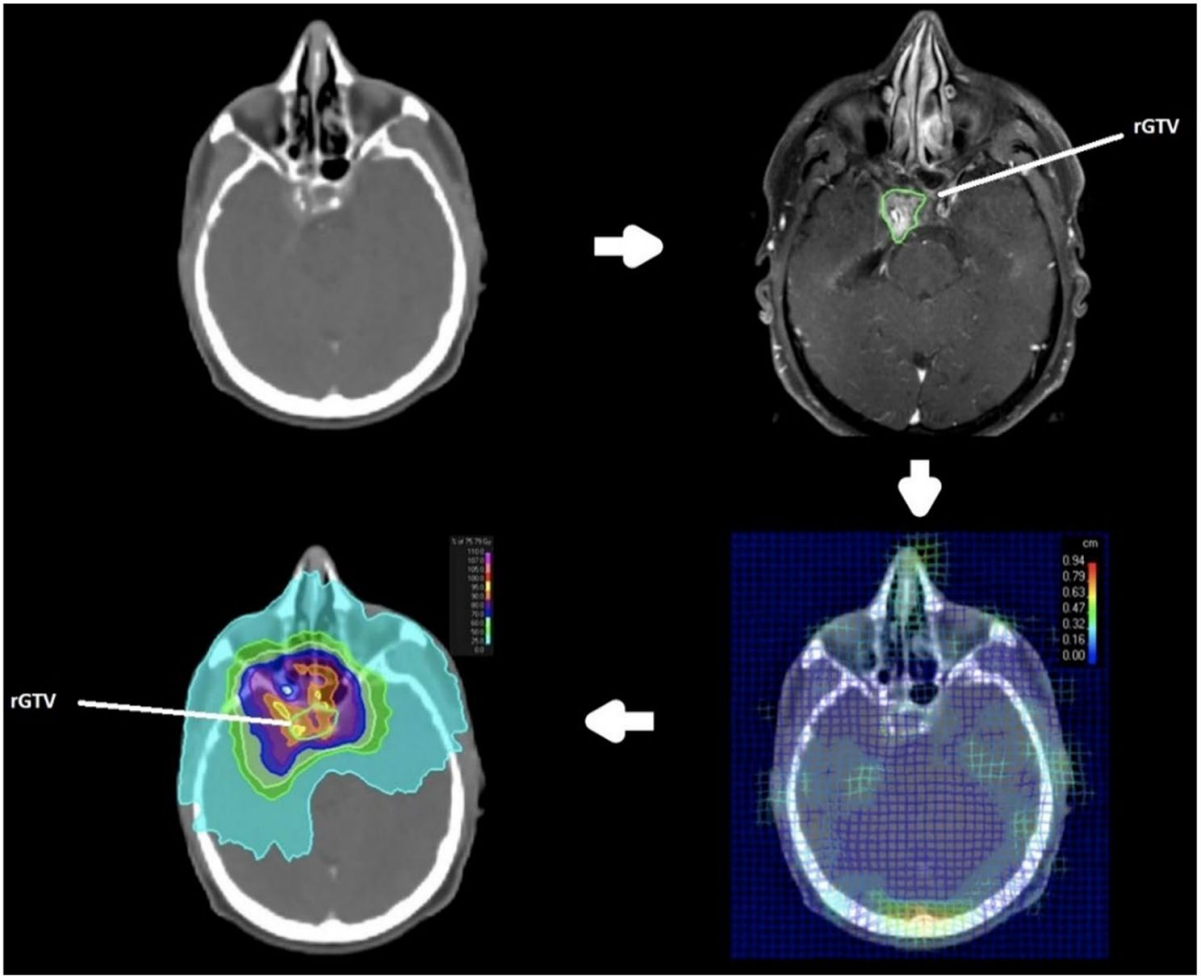
Process for defining recurrent gross tumor volume (rGTV) and applying deformable image registration (DIR). Top left: The initial planning CT (pCT) scan and radiotherapy treatment plan were accessed. Top right: A follow-up CT or MRI scan (rCT/rMRI) capturing tumor recurrence was obtained for rGTV delineation. Bottom right: The recurrence imaging (rCT/rMRI) was aligned with the original pCT scan. Bottom left: The identified rGTV from the rCT/rMRI was deformed onto the co-registered pCT

Recurrent gross tumor volumes (rGTV) were delineated on recurrent CT (rCT) or MRI (rMRI) based on clinical data and radiologic imaging. The original planning CT (pCT) and irradiation plans were retrieved for analysis. Image registration was initially performed using a rigid frame-of-reference approach to align the pCT with the rCT/rMRI, followed by deformable image registration (DIR), with the pCT serving as the reference. DIR was conducted using an anatomically constrained deformation algorithm that incorporated both image intensity and anatomical information. Subsequently, the deformed rGTV was mapped onto the pCT, and a centroid with a 4-mm diameter was defined by applying a 2-mm margin around the central voxel of the rGTV (Fig. 1).

Recurrences were categorized into five distinct types (21, Table 2). The centroid of the deformed rGTV was utilized to identify the originating target volume. The dose administered to 95% of the rGTV (rD95%) was quantified and analyzed in relation to the prescribed dose of the original target volume. In cases where multiple rGTVs were present, the highest classification type was deemed predominant.

**Table 2:** Relapse taxonomy

Relapse type	Relapse volume characteristics
**A** (central high dose)	centroid of rGTV located in PTV70.4 Gy, rD95% ≥ 95% of prescribed dose.
**B** (peripheral high dose)	centroid of rGTV located in PTV70.4 Gy, rD95% < 95% of prescribed dose
**C** (central intermediate/low dose)	centroid of rGTV located in PTV57.6, rD95% ≥ 95% of prescribed dose
**D** (peripheral intermediate/low dose)	centroid of rGTV located in PTV57.6, rD95% < 95% of prescribed dose
**E** extraneous dose	centroid of rGTV outside any PTV

rGTV = recurrent gross tumor volume, PTV = planning target volume, rD95% = dose to 95% of the recurrent gross tumor volume

### Event definitions

Calculation of time-to-event data (OS, LCR, RCR and DCR) was done from the first date of histopathological diagnosis to the date of the last follow-up, death or time of event (local, regional or distant progress) using the Kaplan-Meier method (IBM SPSS statistics version 27). Local treatment failure was defined as recurrence at the primary tumor site, regional failure as recurrence in cervical lymph nodes, and distant failure as recurrence in organs outside the head and neck.

## Results

### Treatment outcome

For all patients, median follow-up was 73 months (range 24–156 months). After 10 years, 31 of the initial 48 patients (65%) were lost to follow-up.

**Fig. 2:**
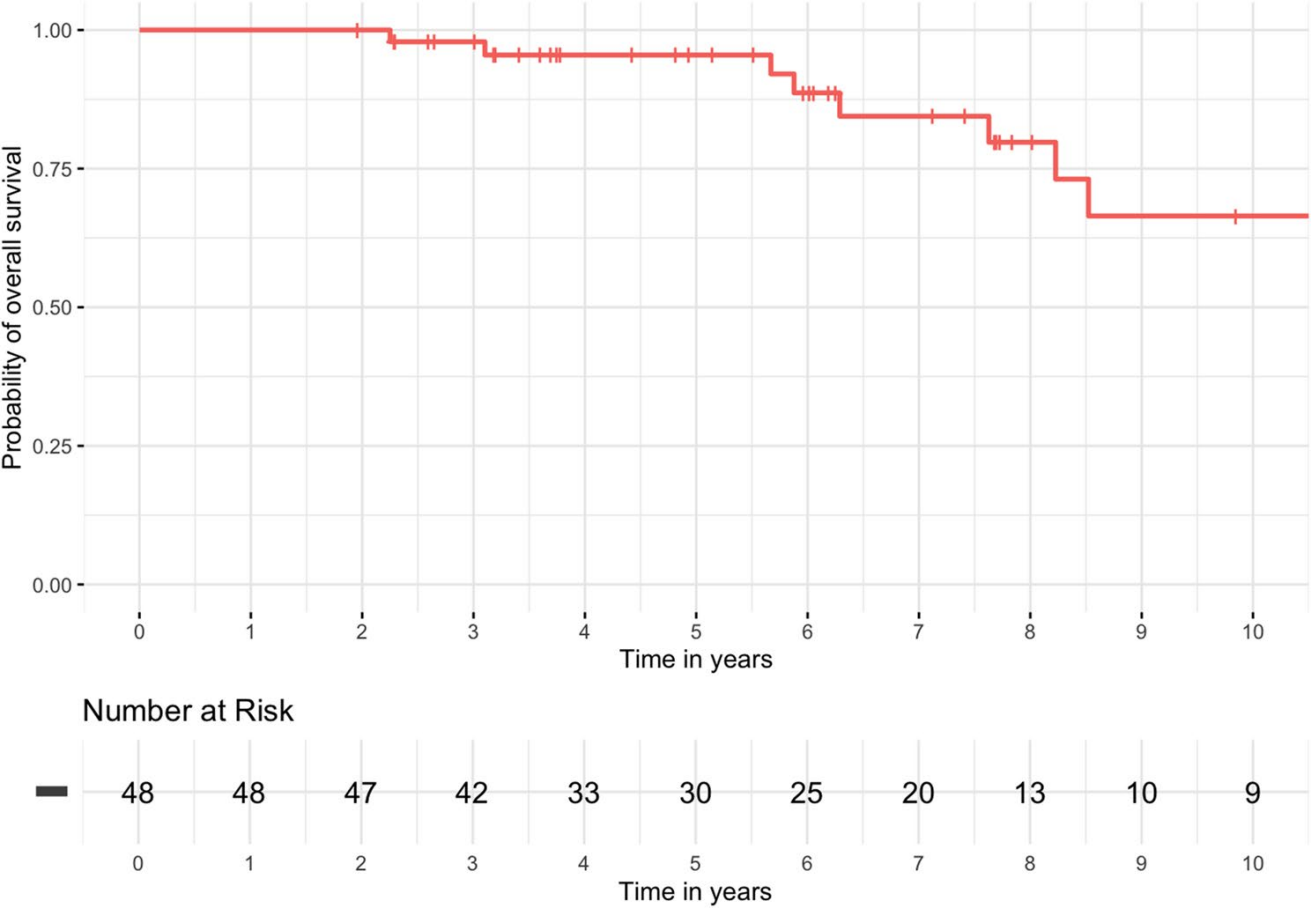
Overall survival

**Fig. 3:**
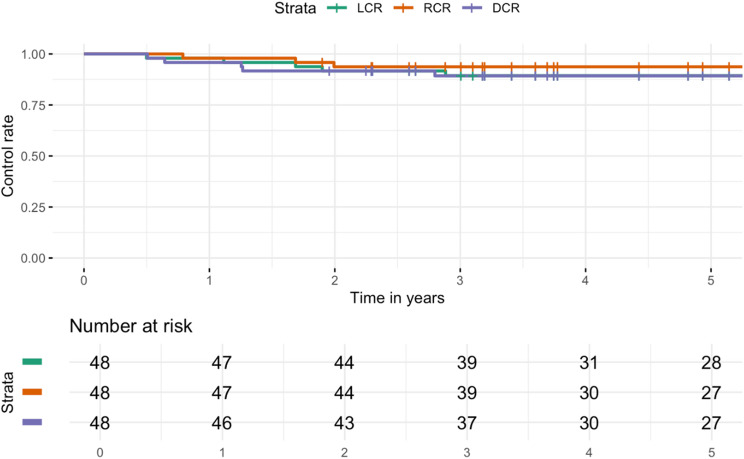
Control rates: Local control rate (LCR), regional control rate (RCR), distant control rate (DCR)

**Fig. 4:**
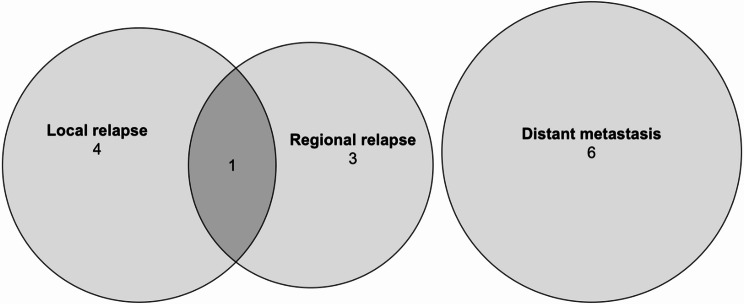
Venn diagram illustrates the distribution of local, regional relapses, and distant metastases

3, 5, and 10-year OS rates were 98%, 96% and 67%. After 2, 3 and 5 years LCR were 92%, 89%, 89%, RCR were 96%, 94%, 94% and DCR rates were 92%, 89%, 89% respectively (Figs. 2 and 3). At the time of their last follow-up examination, 9 patients (19%) had died. Failure after treatment was reported in 14 patients (29%). Treatment failure manifested almost always within the first three years after treatment. Patients who experienced treatment failure showed similar rates of distant metastasis (6 cases, 13%) and local relapse (5 cases, 10%). Nodal failure occurred in 4 patients (8%). Among these cases, one patient (2%) experienced both local and regional recurrence (Fig. 4). The most common types of salvage therapy in cases of treatment failure were chemotherapy (4 cases, 8%) and neck dissection (3 cases, 6%). Two patients (4%) underwent local excision of a solitary distant metastasis, while another two patients (4%) received best supportive care due to distant metastases and poor general condition. Less common salvage therapies included local relapse excision and a combination of local relapse excision and neck dissection, each performed in one case (2%).

### Toxicity

#### Acute toxicity

Acute toxicities comprised adverse events that occurred from the start of radiation until 90 days after. Toxicities were evaluated according to the Common Terminology Criteria of Adverse Events (CTCAE). All patients completed chemoradiotherapy without interruptions. Chemoradiotherapy showed high tolerability without adverse events higher than grade 3. Grade 3 acute toxicity was found in 23 patients (48%) with dysphagia with the need for gastric tube feeding or total parenteral nutrition being the most reported grade 3 adverse event (19 patients, 40%, Table 3). The most common acute toxicities were grade 1 xerostomia (32 patients, 67%), grade 1 dysgeusia (23 patients, 48%) and grade 2 oral mucositis (18 patients, 38%). The mean number of grade 1, 2 and 3 acute toxicity events per patient were 3.2 ((95%- [confidence interval] CI 2.7–3.7), 1.2 (95%-CI 0.9–1.5) and 0.6 (95%-CI 0.4–0.8), respectively (Table 4).

**Table 3:** Acute and late toxicity

Toxicity	CTCAE Grade		
	1	2	3
**Acute**			
Xerostomia	32 (67)	6 (13)	0
Dysgeusia	23 (48)	11 (23)	0
Dysphagia	9 (19)	12 (25)	19 (40)
Mucositis	8 (17)	18 (38)	5 (10)
Nausea	16 (33)	4 (8)	3 (6)
Lymphedema	5 (10)	0	0
Dermatitis radiation	8 (17)	6 (13)	2 (4)
Tympanic effusion	0	8 (17)	0
Fatigue	4 (8)	1 (2)	0
**Late**			
Xerostomia	31 (65)	10 (21)	0
Dysgeusia	31 (65)	1 (2)	0
Dysphagia	11 (23)	2 (4)	1 (2)
Mucositis	1 (2)	0	0
Nausea	0	0	0
Lymphedema	8 (17)	0	0
Tympanic effusion	0	7 (15)	0
Fatigue	6 (13)	1 (2)	0
Blood-brain barrier damage	1 (2)	0	0
Osteonecrosis	0	0	2 (4)

**Table 4:** Mean number of CTCAE v5.0 toxicity events per patient

	Mean (95%-CI)	Patients (%)
**Acute**		
CTC grade I	3.2 (2.7–3.7)	46 (96)
CTC grade II	1.2 (0.9–1.5)	36 (75)
CTC grade I-II	4.4 (3.9–4.9)	47 (98)
CTC grade III	0.6 (0.4–0.8)	23 (48)
**Late**		
CTC grade I	2.3 (1.8–2.7)	42 (88)
CTC grade II	0.3 (0.1–0.5)	12 (25)
CTC grade I-II	2.6 (2.1–3.1)	42 (88)
CTC grade III	0.1 (0-0.2)	3 (6)

*CTCAE* Common Terminology Criteria of Adverse Events, *CI* Confidence interval; Comment: Acute toxicity that continued in the late phase was counted as both

acute and late toxicity. If a patient had more than one toxicity event, only the highest toxicity grade was counted once in the total count category

#### Late toxicity

Late toxicity included adverse events reported more than 90 days after initiation of chemoradiotherapy. No patient presented grade 4 or 5 late toxicity. Most of the described grade 3 acute adverse events had resolved until the first or second follow-up examination after treatment. Only 1 patient (2%) suffered from grade 3 late dysphagia. The mean number of grade 1, 2 and 3 late toxicity events per patient were 2.3 ((95%-CI 1.8–2.7), 0.3 (95%-CI 0.1–0.5) and 0.1 (95%-CI 0.0-0.2), respectively.

Regarding brain injury, one patient showed an asymptomatic blood-brain barrier damage grade 1 in the right temporal lobe 2 years after initiation of chemoradiotherapy. There was no medical intervention necessary. Moreover, two patients showed grade 3 osteoradionecrosis which was successfully treated by an osteotomy. The most common late adverse events comprised grade 1 xerostomia and dysgeusia (31 patients, 65%), grade 1 dysphagia (11 patients, 23%) and grade 1 lymphedema (8 patients, 17%). Acute and late toxicity in detail is shown in Tables 3 and 4.

### Dose-level-specific patterns of locoregional recurrence

**Fig. 5:**
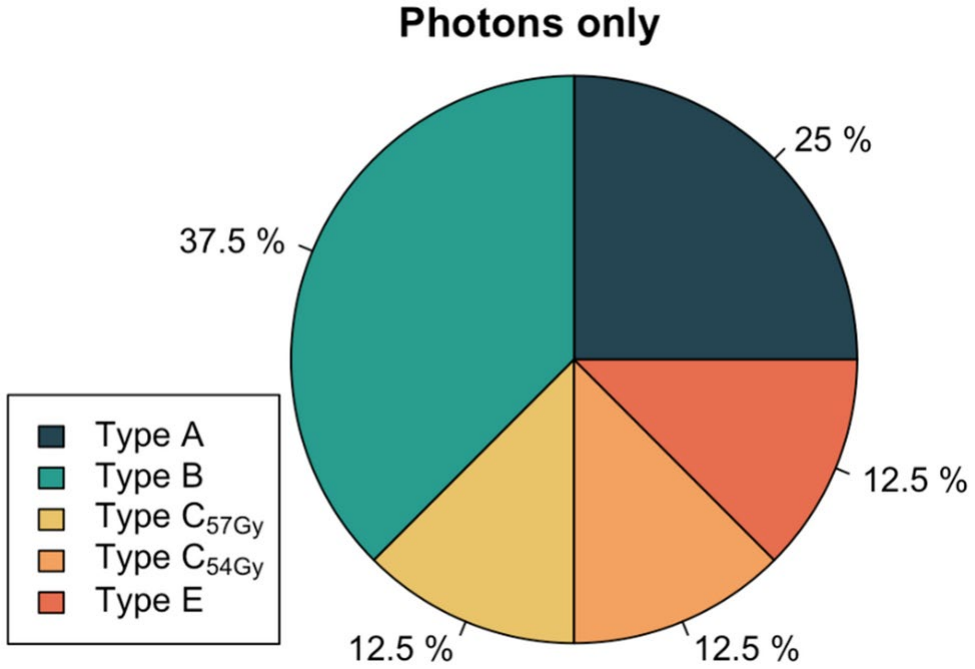
Pie chart illustrating the distribution of the predominant recurrence typologies, including the specific locations of type C low-dose recurrences. Recurrences *n* = 8 (100%)

Among the four evaluable patients with isolated local recurrence, three with nodal failure alone, and one with locoregional relapse, a total of five cases (63%) were classified as high-dose failures, comprising two type A and three type B failures. In contrast, three cases (37%) were non-high-dose failures, with two categorized as type C low-dose and one as type E. Specifically examining non-central high-dose failures, two of the three type B failures resulted from insufficient dose coverage in PTV1 due to its close anatomical proximity to the optical system. The single type E extraneous dose failure corresponded to a nodal metastasis located in the parotid gland (level VIII). Visual representations of recurrence patterns are provided in Figs. 4 and 5.

## Discussion

The treatment of NPC with IMRT combined with weekly concomitant cisplatin resulted in encouraging outcomes, with an OS rate of 96%, a LCR rate of 89%, a RCR of 94%, and a DCR of 89% at five years. The majority of patients presented with advanced-stage disease, with 88% classified as UICC stage III or higher (Table 1). Chemoradiotherapy was associated with moderate toxicity, with no treatment-related adverse events exceeding CTCAE grade 3. Approximately half of the patients experienced at least one acute grade 3 adverse event, which had resolved in nearly all cases within 90 days post-treatment. Late toxicity was minimal, with only 6% of grade 3 adverse events persisting beyond this period (Tables 3 and 4).

According to current guidelines, one option for first-line therapy for locally advanced NPC consists of IMRT administered at a total dose of 70 to 72 Gy to the primary tumor, combined with concomitant chemotherapy using weekly cisplatin at a dose of 40 mg/m² body surface area [5–7]. Our treatment outcomes align with findings from recent literature, demonstrating the efficacy of IMRT with OS rates reported at 92% at two years, 86% at three years, and 79.6% at five years [12, 26, 27]. In light of these benchmarks, our findings suggest particularly favorable outcomes, with a five-year OS rate of 96%.

Based on the findings of our study, acute and late toxicities associated with chemoradiotherapy demonstrated high tolerability, with no adverse events exceeding grade 3. In comparison to previous studies, our results are in line with the reported toxicity profiles of chemoradiotherapy for nasopharyngeal carcinoma. For instance, the phase III Intergroup 0099 study demonstrated a higher incidence of grade 3 and 4 acute toxicities, particularly hematologic and gastrointestinal, with concurrent chemoradiotherapy compared to radiotherapy alone [28]. Similarly, the MAC-NPC meta-analysis reinforced the superior efficacy of chemoradiotherapy in nasopharyngeal carcinoma but highlighted an increased risk of acute toxicities, particularly mucositis and hematologic complications [29]. Our study aligns with these findings but suggests a relatively lower frequency of severe acute toxicity events, which may be attributed to optimized supportive care and proactive multidisciplinary management. Additionally, recent studies such as Tang et al. indicated that the omission of chemotherapy in low-risk patients significantly reduced acute grade 3 or 4 toxicities, particularly hematologic and gastrointestinal side effects [30]. Furthermore, our data showed a low incidence of grade 3 late toxicities, with only one case of grade 3 dysphagia, which is lower than previous reports, including the study by Zhang et al., which highlighted a higher rate of late adverse effects following the addition of induction chemotherapy [31]. Notably, our findings suggest that while chemoradiotherapy remains the standard of care, continued refinement in toxicity management is essential to minimize treatment-related morbidity without compromising efficacy.

In our study, distant metastasis was one of the most common pattern of failure following treatment, occurring in 6 patients (13%). The very limited use of adjuvant chemotherapy in this cohort reflects the treatment era (2005–2019) and prevailing guidance at our center. At that time, ESMO recommendations allowed concurrent chemoradiotherapy alone as a first-line option for stage III–IVA disease (level IA), whereas NCCN already favored induction chemotherapy followed by chemoradiotherapy and assigned chemoradiotherapy alone only a category 2B recommendation [8]. Moreover, adjuvant chemotherapy after chemoradiotherapy was not routinely pursued following the negative randomized trial of cisplatin/5-fluorouracil [9]. More recent evidence supporting maintenance capecitabine and the broader adoption of induction chemotherapy postdates our accrual window and has since informed our current practice [5–7, 31–35]. Collectively, these findings underscore the central role of optimized systemic therapy in NPC and align with contemporary data supporting induction strategies and, when induction is not given, consideration of adjuvant therapy.

To our knowledge, this is the first study to apply the locoregional recurrence pattern analysis methodology proposed by Mohamed et al. to patients with nasopharyngeal carcinoma treated with chemoradiotherapy [21]. Our study identified that among the evaluable cases of recurrence, high-dose failures accounted for 63%, with type B failures representing the majority. These findings align with previous research emphasizing that high-dose failures constitute the predominant pattern of recurrence in NPC treated with IMRT [36]​. Notably, the occurrence of type B failures in our cohort appears to be partially attributable to insufficient dose coverage in PTV1 due to the proximity of the target volume to the optical system, which constrained dose escalation. Similar challenges in dose optimization near critical structures have been documented in the literature, underscoring the need for advanced radiotherapy techniques to enhance dose conformity while minimizing toxicity [15, 17]​​. A potential strategy to address this limitation is the integration of carbon ion radiotherapy as a boost to IMRT, leveraging its superior biological effectiveness and enhanced conformity to improve local control while sparing adjacent organs at risk [37–42]​. Furthermore, our findings highlight the need for refined target volume delineation, as anatomical constraints may necessitate individualized adaptations to treatment planning, as suggested in prior studies advocating for precision in clinical target volume definitions [16]. Newly released consensus guidelines for target volume delineation in radiotherapy for nasopharyngeal carcinoma from CSTRO, CACA, CSCO, HNCIG, ESTRO, and ASTRO have been published, and their clinical impact will become clearer as they are implemented and evaluated in prospective cohorts and real-world practice [18, 19]. Future research should explore the efficacy of combined IMRT and carbon ion boost in mitigating high-dose failures, particularly in regions with dose limitations due to organ-at-risk constraints.

When interpreting the findings of this study, several limitations must be considered. The retrospective design inherently limits the strength of the conclusions, as no comparison was made with a prospective randomized trial. The very limited use of adjuvant chemotherapy in our cohort - reflecting 2005–2019 practice patterns and contemporaneous ESMO guidance - may have influenced distant failure rates and reduces comparability with contemporary, induction-based standards. A further limitation is the proportion of patients with unknown tumor EBV status. This reflects historical practice during the early enrolment period (from 2005) in a non-endemic, single-center European setting, when routine EBV testing was not yet widely recommended by contemporary guidelines and only became formalized later. Although the study includes a long follow-up period of up to 156 months, a substantial proportion of patients (31 out of 48; 65%) were lost to follow-up, and the overall number of patients was relatively small. This limitation reflects the low incidence of nasopharyngeal carcinoma in non-endemic regions compared with Asia. However, for a single-center experience in Europe, the sample size of 48 patients remains reasonable. The uniform treatment protocol applied across all cases ensures a homogeneous cohort, thereby strengthening the internal validity of our findings. The study also provides valuable insights by applying a more comprehensive framework for classifying failure patterns.

## Conclusion

IMRT combined with weekly cisplatin demonstrated excellent efficacy and tolerability in nasopharyngeal carcinoma, with a five-year overall survival rate of 96% and no adverse events exceeding grade 3. The observation that distant metastasis represents one of the predominant patterns of treatment failure reinforces the critical role of effective systemic therapy in the comprehensive management of nasopharyngeal carcinoma. High-dose failures, particularly type B, were linked to dose constraints near critical structures. Exploring advanced radiotherapy techniques, e.g. integrating carbon ion therapy, may improve dose conformity and local control.

## Data Availability

No datasets were generated or analysed during the current study.

## Abbreviations

3D-RT: Three-dimensional radiotherapy
ACC: Adenoid cystic carcinoma
AJCC: American Joint Committee on Cancer
CT: Computed tomography
CTCAE: Common Terminology Criteria for Adverse Events
CTV: Clinical target volume
DCR: Distant control rate
DIR: Deformable image registration
EBV: Epstein-Barr virus
ESMO: European Society for Medical Oncology
IMRT: Intensity-Modulated Radiotherapy
LC: Local control
LCR: Local control rate
MRI: Magnetic resonance imaging
NCCN: National Comprehensive Cancer Network
NPC: Nasopharyngeal carcinoma
OS: Overall survival
pCT: Planning computed tomography
PFS: Progression-free survival
PTV: Planning target volume
RCR: Regional control rate
rCT: Recurrent computed tomography
rGTV: Recurrent gross tumor volume
rMRI: Recurrent magnetic resonance imaging
SIB: Simultaneous integrated boost
UICC: Union for International Cancer Control
